# Risk of mortality following discharge from Critical Care

**DOI:** 10.23889/ijpds.v1i1.330

**Published:** 2017-04-19

**Authors:** Angharad Walters, Ronan Lyons, Damon Berridge

**Affiliations:** 1 The Farr Institute, Swansea University

## Objectives

Critical care provides specialist treatment for patients with life-threatening illnesses and injuries. Outcomes data are fundamental to identify where resources need to be focused to provide safer care; we can determine long term survival after critical care and identify the risk factors of mortality by linking the critical care data with a mortality register, thereby building on previous survival reporting which is constrained to mortality within the admission. The objective is to identify the factors that increase the risk of mortality following discharge from critical care.

## Approach

Anonymised critical care data reported from 1st April 2006 are held within the Secure Anonymised Information Linkage (SAIL) databank and will be linked with ONS mortality data, inpatients data and the Welsh Demographic Service dataset. Details of patient care during the critical care admission such as duration of level 2/3 support, the organs supported and discharge details such as the time of day, along with patient demographics such as age, sex and socioeconomic factors are available for analysis. Adult Welsh patients alive at the discharge date and with a discharge date from critical care on or before 31.12.2013 will be followed up until 31.12.2014, outward migration or death.

## Results

Survival analysis techniques will be used to determine the risk of death at certain time points after discharge from critical care and to identify the risk factors associated with mortality. 

## Conclusion

Determining the factors associated with mortality will allow patients at highest risk of death to be identified. If these risk factors are modifiable, then preventive interventions can be introduced to reduce the number of avoidable deaths.

